# Inline spectrometer for shot-by-shot determination of pulse energies of a two-color X-ray free-electron laser

**DOI:** 10.1107/S1600577515020196

**Published:** 2016-01-01

**Authors:** Kenji Tamasaku, Yuichi Inubushi, Ichiro Inoue, Kensuke Tono, Makina Yabashi, Tetsuya Ishikawa

**Affiliations:** a RIKEN SPring-8 Center, 1-1-1 Kouto, Sayo-cho, Sayo-gun, Hyogo 679-5148, Japan; b JASRI, 1-1-1 Kouto, Sayo-cho, Sayo-gun, Hyogo 679-5198, Japan; c Graduate School of Frontier Sciences, The University of Tokyo, 5-1-5 Kashiwanoha, Kashiwa, Chiba 277-8561, Japan

**Keywords:** XFEL, two color, pump and probe, spectrometer

## Abstract

An inline spectrometer has been developed to monitor shot-by-shot pulse energies of a two-color X-ray beam. A thin film of diamond allows inline operation with minimum absorption. The absolute pulse energy for each color is determined by the inline spectrometer combined with a total pulse-energy monitor. A negative correlation is found between the two-color pulse energies.

## Abstract 2

An inline spectrometer has been developed to monitor each pulse energy of a two-color X-ray beam.

## 1. Introduction   

Recently, two-color operation (Hara *et al.*, 2013 ▸; Lutman *et al.*, 2014 ▸) of the X-ray free-electron laser (XFEL) has been realised at SACLA (Ishikawa *et al.*, 2012 ▸) and LCLS (Emma *et al.*, 2010 ▸). These unique sources should open up advanced XFEL applications, such as X-ray pump/X-ray probe experiments. The data analysis requires information about each pulse energy of the two colors. In addition, the pulse energies, which fluctuate shot by shot, must be monitored during the experiment. Since the total pulse-energy monitor assumes a single-color beam (Tono *et al.*, 2013 ▸; Emma *et al.*, 2010 ▸), a new method is required to measure the spectrally resolved pulse energy. A bent-crystal spectrometer (Zhu *et al.*, 2012 ▸) may be used for smaller photon-energy separations, *e.g.* several eV as in the case of LCLS. However, there is no method for a keV-separated two-color beam. In this short communication we report absolute and shot-by-shot monitoring of two-color pulse energies at 8.05 and 9.1 keV using a polycrystalline inline spectrometer.

## 2. Experimental setup   

**Figure 1:**
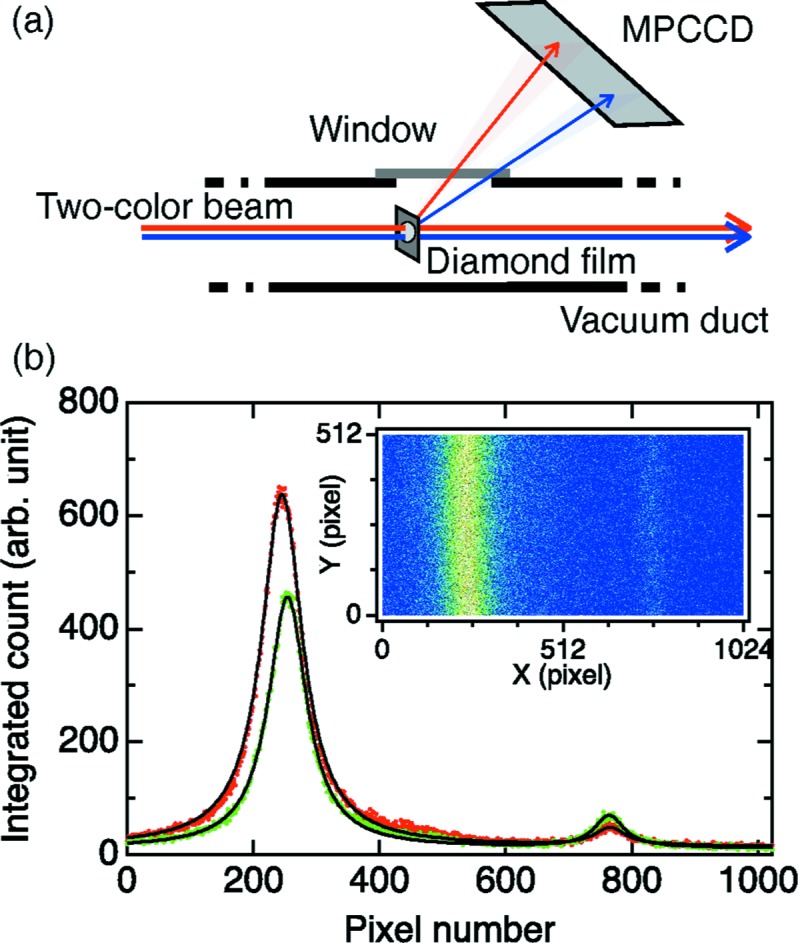
(*a*) Schematic of the inline spectrometer. (*b*) Measured single-shot spectra. Inset: a typical diffraction image taken by the MPCCD.

Fig. 1(*a*) ▸ shows a schematic diagram of the inline spectrometer. The basic design is the same as for the beamline wavelength monitor (Tono *et al.*, 2013 ▸), but is modified to be compact and portable. The two-color beam is intercepted by a thin polycrystalline diamond film. A 15 µm-thick nano-diamond film was CVD-grown on a silicon substrate, and then the substrate was removed by etching (Tono *et al.*, 2011 ▸). The transmittance is calculated to be more than 97% for photon energies above 8 keV (Henke *et al.*, 1993 ▸), enabling inline monitoring. A small part of the beam is diffracted kinematically into different directions according to the Bragg condition. A multi-port CCD (MPCCD) detector (Kameshima *et al.*, 2014 ▸) captures the diffracted beam image at the repetition rate of SACLA (30 Hz). The film-to-MPCCD distance is adjustable to obtain a proper separation between the two beam images.

The performance of the inline spectrometer was evaluated at beamline BL3 of SACLA (Ishikawa *et al.*, 2012 ▸). The two-color beam was produced by the so-called split-undulator scheme (Hara *et al.*, 2013 ▸). The relative pulse-energy ratio can be tuned by changing the numbers of undulator segments. In this experiment, six upstream segments of the undulator were used for a weak beam at 9.1 keV, and the remaining fifteen were used for a strong beam at 8.05 keV. The inline spectrometer must be placed downstream of the final aperture, which was a four-jaw slit in this experiment, because the source-to-aperture distance, and therefore the solid angle accepted by the aperture, depends on the color in the split-undulator scheme. This is the reason why we have constructed the present portable inline spectrometer.

## 3. Result and discussion   

The inset of Fig. 1(*b*) ▸ shows the typical image of the diamond 220 diffraction. The photon energy disperses along the *X* direction. The *Y* direction corresponds to the spatial distribution. Strictly speaking, the diffraction image is an arc, which is a part of the Debye–Scherrer cone. However, the effect of the curvature is minor compared with the broad peak width, and is ignored below. The count of each pixel is added vertically to obtain the spectrum. Fig. 1(*b*) ▸ shows spectra obtained in different shots. The peak heights change shot by shot, while the peak photon energies are almost fixed.

The spectrum can be reproduced well by a sum of two Lorentzian functions and a constant,
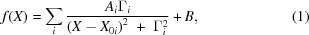
where *A* is the weight, Γ is the width and 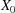 is the center pixel of the peak. The constant *B* represents the background due to air scattering. The peak widths are estimated to be about 150 eV. This is larger than the spectral bandwidth of ∼50 eV measured separately by the beamline monochromator. The spread is accounted for by the grain size of the diamond film. The width imposes the lower limit of the photon-energy separation between the two colors, which we estimate as about 400 eV. Nevertheless, the peak position can be determined with a nominal resolution of 2.1 eV, which is given by the pixel size of 50 µm and the film-to-MPCCD distance.

The readout of the MPCCD is proportional to both the photon energy and the photon number (Kameshima *et al.*, 2014 ▸), so that the fitting parameter, *A*, is proportional to the pulse energy. However, 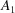 and 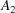 cannot be compared without correcting the photon-energy-dependent factors, such as the transmittance of air, *T*, the structure factor, *F*, and the quantum efficiency of the MPCCD, *Q*. Correcting these factors, the absolute pulse energy may be given by
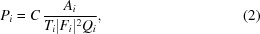
where 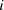 = 1, 2 denotes the color, and *C* is a common conversion constant. The relative pulse-energy variation of each color can be determined without knowledge of *C*. The structure factor appears as 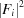 because of the kinematical nature of diffraction.

If we performed an additional measurement in the single-color mode, we could determine *C* directly by a total pulse-energy monitor. Here, we discuss another approach without changing the color mode. In general, the sensitivity of the total pulse-energy monitor depends on the photon energy. When a pulse-energy monitor measures the two-color beam, the output signal, *S*, may be given by a sum of that for each color:
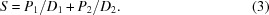
Here, *D* is the known conversion coefficient of the total pulse-energy monitor. Combining (3) with (2), *C* can be determined from 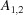 and *S*, which are to be measured experimentally by the inline spectrometer and the total pulse-energy monitor, respectively.

In the present experiment, we used a beam-position monitor (BPM) as the total pulse-energy monitor (Tono *et al.*, 2013 ▸). The BPM consists of a thin diamond film and quadrant photodiodes, which measure the backscattered X-rays by the film. The pulse energy is determined from *S*, the sum of the four charges measured by the photodiodes. The output of the photodiode is not the current but the charge, because the XFEL is a pulsed source. The conversion coefficient of the BPM, *D*, is calibrated to a cryogenic radiometer (Kato *et al.*, 2012 ▸).

**Figure 2:**
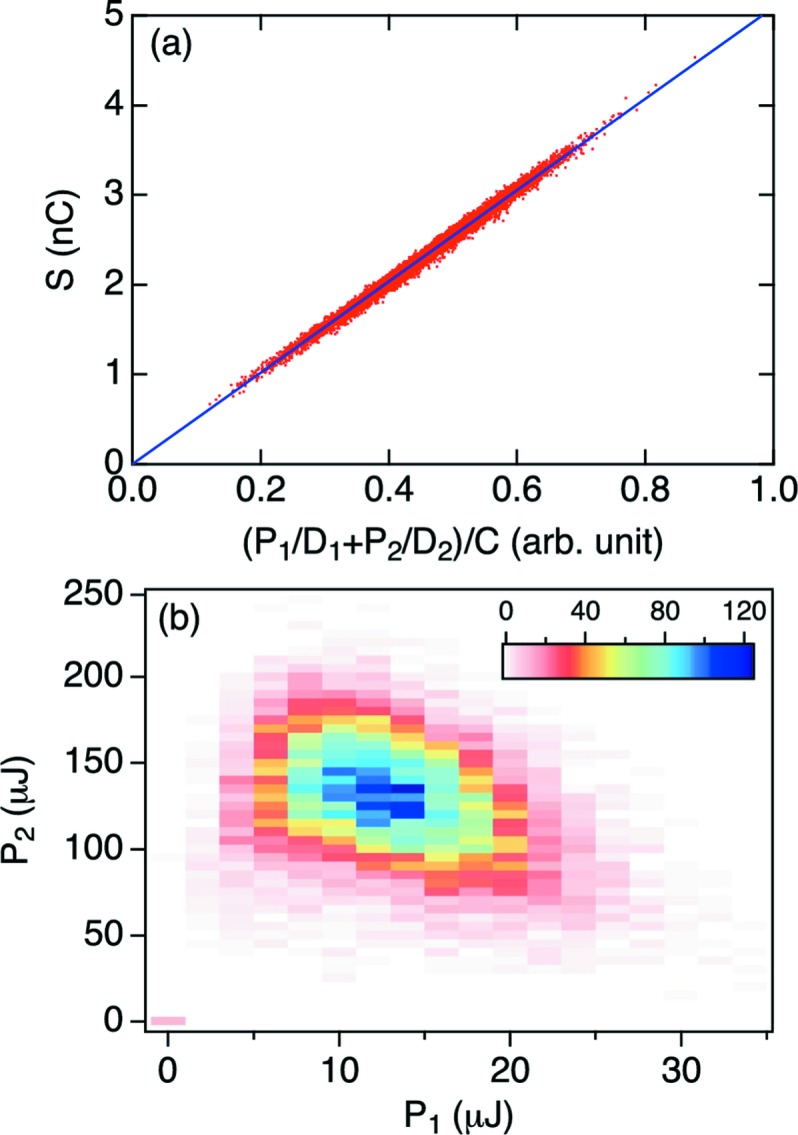
(*a*) Measured output signal of the total pulse-energy monitor (BPM) plotted as a function of the calculated one from the spectrum analysis of the inline spectrometer. The solid line represents the best fitting with a linear function. (*b*) Distribution of two-color pulse energies, 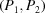. The color bar indicates the frequency.

Fig. 2(*a*) ▸ shows the relation between the measured *S* by the BPM and the calculated *S* from 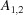 using (2) and (3). The data points lie in the vicinity of a line crossing the origin, indicating the validity of our analysis. The conversion coefficient is estimated to be 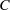 = 5.0883 ± 0.00162 from the slope of the linear fitting. Now, the shot-by-shot pulse energy for each color can be calculated using (2). The 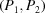 distribution of 10000 shots is plotted in Fig. 2(*b*) ▸. A negative correlation is found between 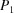 and 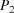, which is considered to be a natural consequence of the fact that the two colors originate from the same electron beam.

Finally, we discuss the error of 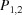. The error arises from the fitting parameters, *C* and 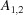, and the conversion coefficients, 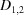. The average photon numbers at the peak are 4.2 photons per pixel for 8.05 keV and 0.25 for 9.1 keV. The shot noise dominates the fitting error of 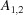, while the readout noise of the MPCCD is negligible. The uncertainty of 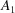 and 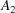 are estimated to be 3.1% and 7.5%, respectively. The larger error for 9.1 keV is due to the weaker signal. The relative uncertainty of 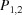 is determined by that for 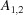, because the error of *C* is much smaller. The uncertainty of the absolute pulse energy depends on that of the total pulse-energy monitor as well. When we adapt 2.5% evaluated at 9.6 keV to that of 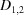 (Kato *et al.*, 2012 ▸), we estimate the uncertainty of 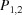 to be 4.0% for 8.05 keV and 8.0% for 9.1 keV.

## 4. Conclusion   

In conclusion, we have developed and operated successfully an inline spectrometer to monitor the relative two-color pulse energies of SACLA shot-by-shot. Furthermore, using a calibrated BPM, the absolute two-color pulse energies are determined, which will serve for quantitative analysis of two-color XFEL experiments. Although the photon-energy resolution of the present inline spectrometer is not enough for smaller separations, it can be improved by increasing the grain size of the diamond film.

## References

[bb1] Emma, P. *et al.* (2010). *Nat. Photon.* **4**, 641–647.

[bb2] Hara, T., Inubushi, Y., Katayama, T., Sato, T., Tanaka, H., Tanaka, T., Togashi, T., Togawa, K., Tono, K., Yabashi, M. & Ishikawa, T. (2013). *Nat. Commun.* **4**, 2919. 10.1038/ncomms3919 24301682

[bb3] Henke, B. L., Gullikson, E. M. & Davis, J. C. (1993). *At. Data Nucl. Data Tables*, **54**, 181–342.

[bb4] Ishikawa, T. *et al.* (2012). *Nat. Photon.* **6**, 540–544.

[bb5] Kameshima, T., Ono, S., Kudo, T., Ozaki, K., Kirihara, Y., Kobayashi, K., Inubushi, Y., Yabashi, M., Horigome, T., Holland, A., Holland, K., Burt, D., Murao, H. & Hatsui, T. (2014). *Rev. Sci. Instrum.* **85**, 033110. 10.1063/1.4867668 24689567

[bb6] Kato, M., Tanaka, T., Kurosawa, T., Saito, N., Richter, M., Sorokin, A. A., Tiedtke, K., Kudo, T., Tono, K., Yabashi, M. & Ishikawa, T. (2012). *Appl. Phys. Lett.* **101**, 023503.

[bb7] Lutman, A. A., Decker, F.-J., Arthur, J., Chollet, M., Feng, Y., Hastings, J., Huang, Z., Lemke, H., Nuhn, H.-D., Marinelli, A., Turner, J. L., Wakatsuki, S., Welch, J. & Zhu, D. (2014). *Phys. Rev. Lett.* **113**, 254801. 10.1103/PhysRevLett.113.254801 25554887

[bb8] Tono, K., Kudo, T., Yabashi, M., Tachibana, T., Feng, Y., Fritz, D., Hastings, J. & Ishikawa, T. (2011). *Rev. Sci. Instrum.* **82**, 023108. 10.1063/1.3549133 21361574

[bb9] Tono, K., Togashi, T., Inubushi, Y., Sato, T., Katayama, T., Ogawa, K., Ohashi, H., Kimura, H., Takahashi, S., Takeshita, K., Tomizawa, H., Goto, S., Ishikawa, T. & Yabashi, M. (2013). *New J. Phys.* **15**, 083035.

[bb10] Zhu, D., Cammarata, M., Feldkamp, J. M., Fritz, D. M., Hastings, J. B., Lee, S., Lemke, H. T., Robert, A., Turner, J. L. & Feng, Y. (2012). *Appl. Phys. Lett.* **101**, 034103.

